# Misdiagnosis of invasive pulmonary fungal disease caused by drug-resistant *Candida tropicalis* as airway neoplasm: case report and literature review

**DOI:** 10.3389/fmed.2026.1800622

**Published:** 2026-04-10

**Authors:** Yuanyuan Liu, Yingting Peng, Jia Nie, Yi Li, Xiaolong Li, Yinhe Feng

**Affiliations:** 1 Department of Respiratory and Critical Care Medicine, Deyang People’s Hospital, Affiliated Hospital of Chengdu University of TCM, Deyang, China; 2 Department of Pathology, Deyang People’s Hospital, Affiliated Hospital of Chengdu University of TCM, Deyang, China

**Keywords:** *Candida tropicalis*, case report, fluconazole resistance, invasive pulmonary fungal disease, malignancy

## Abstract

Invasive pulmonary fungal disease (IPFD) is often missed or misdiagnosed. Here we report a rare case of IPFD caused by multidrug-resistant *Candida tropicalis*, which was initially misdiagnosed at another hospital as recurrent pulmonary lymphoma because the patient had a history of lymphoma. Histopathology, sputum culture, and next-generation sequencing of bronchoalveolar lavage fluid at our hospital indicated that IPFD was caused by *C. tropicalis*. The patient did not respond to fluconazole but showed substantial improvement with caspofungin. We discovered a cutaneous malignancy, which we determined to be the most likely cause of the immunosuppression that facilitated invasive *C. tropicalis* infection. Our case highlights that IPFD caused by *C. tropicalis* can show unusual manifestations, which can lead to misdiagnosis as malignancy; that response to antifungals should be monitored carefully because of the risk of resistance in circulating *C. tropicalis* strains; and that underlying causes of the immunosuppression that led to *C. tropicalis* infection should be explored, which may uncover deeper problems.

## Introduction

Invasive pulmonary fungal disease (IPFD) occurs predominantly in individuals who are immunocompromised because of hematological disorders or solid organ transplantation. The fungus invades lung tissue and the blood, which can lead to complications in other parts of the body. Relatively few cases of IPFD involve the fungus *Candida tropicalis*, but 55%–60% of such cases result in death (1). *C. tropicalis*-associated IPFD typically manifests on chest imaging as patchy or diffuse infiltrative shadows if the lungs have been infected directly, or as multiple nodular or mass-like shadows if the fungus arrived in the lungs through the blood (2, 3). In rare cases, it can manifest as invasive aspergilliform neoplasms in the airway.

Invasive pulmonary fungal disease is often missed or misdiagnosed, especially when its manifestations or the patient’s characteristics are atypical (4). Here we report a rare case of IPFD in which *C. tropicalis* concurrently caused invasive pneumonia and invasive tracheobronchitis. The patient was presented to a local hospital with endobronchial neoplasm, which in light of her history of lymphoma was misdiagnosed as recurrent pulmonary lymphoma. After a definitive diagnosis at our hospital, the patient failed to respond to the antifungal fluconazole but responded well to caspofungin, both of which are routinely used to treat *C. tropicalis* infections. The immunosuppression that facilitated the infection was likely due to an undiagnosed cutaneous malignancy. The characteristics of this case highlight the diverse manifestations of *C. tropicalis*-induced IPFD.

## Case presentation

A 58-year-old Chinese woman was presented at our hospital with complaints of recurrent coughing and expectoration during the preceding 6 months, accompanied by fever and dyspnea for the preceding 10 days. She reported no chest pain, hemoptysis, hot flushes or night sweats. She had a history of non-Hodgkin lymphoma, for which she had received standard radio- and chemotherapy, which ended more than 2 years before her admission to our hospital. She denied previous or current smoking or alcohol consumption, and she reported no family history of hereditary disease.

Fiber-optic bronchoscopy at another hospital had revealed a yellowish-white neoplasm obstructing the lumen of the left main bronchus. Bronchoalveolar lavage fluid (BALF) tested positive for Epstein-Barr virus, with no malignant tumor cells detected among the exfoliated cells. Her condition did not improve after 7 days of cefoxitin therapy, so she was referred to our institution with the tentative diagnosis of pulmonary lymphoma recurrence complicated by infection.

On admission to our hospital, the patient appeared lethargic and showed a body temperature of 36.8 °C; pulse, 84 beats per minute; respiratory rate, 21 breaths per minute; blood pressure, 107/55 mmHg; and oxygen saturation with room air, 94%. Moist rales were audible in both lungs. No abnormalities were found in complete blood count, arterial blood gas analysis, infection screening (including hepatitis B, hepatitis C, syphilis and HIV), or assays of B-type natriuretic peptide, β-D-glucan, troponin I, electrolytes, renal or hepatic function, or coagulation activity. Yeast-like spores grew from sputum smears, which were identified as *Candida tropicalis*.

**FIGURE 1:**
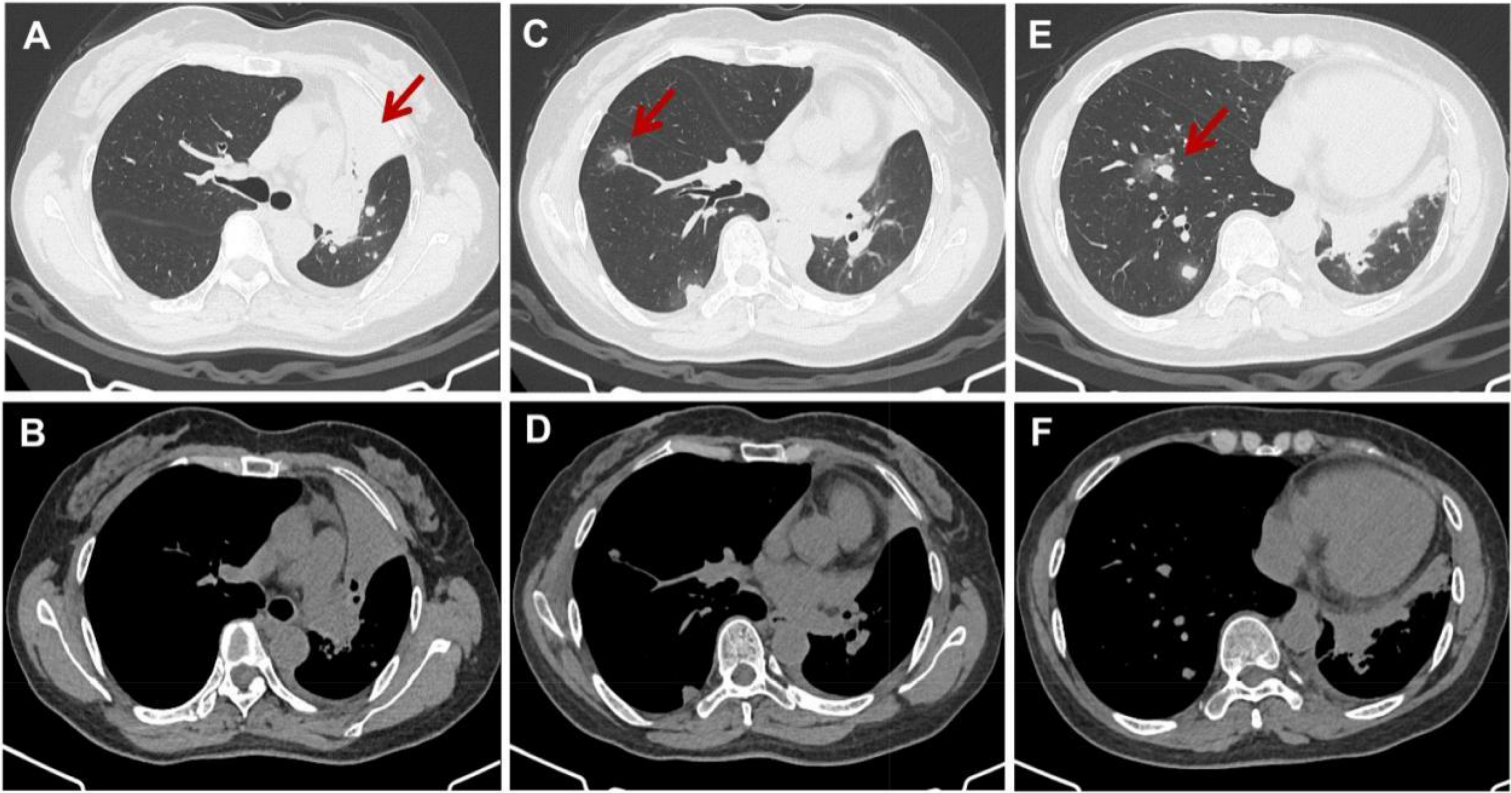
Computed tomography of the chest at admission. Images reveal **(A)** consolidation of the left upper lobe with atelectasis (red arrow), as well as **(C,E)** multiple bilateral patchy opacities, nodules, and masses with a peripheral halo sign (red arrows). The corresponding mediastinal images are shown in the lower row. **(B,D,F)** Represent the mediastinal window images corresponding to the layers of panels **(A,C,E)** respectively.

**FIGURE 2:**
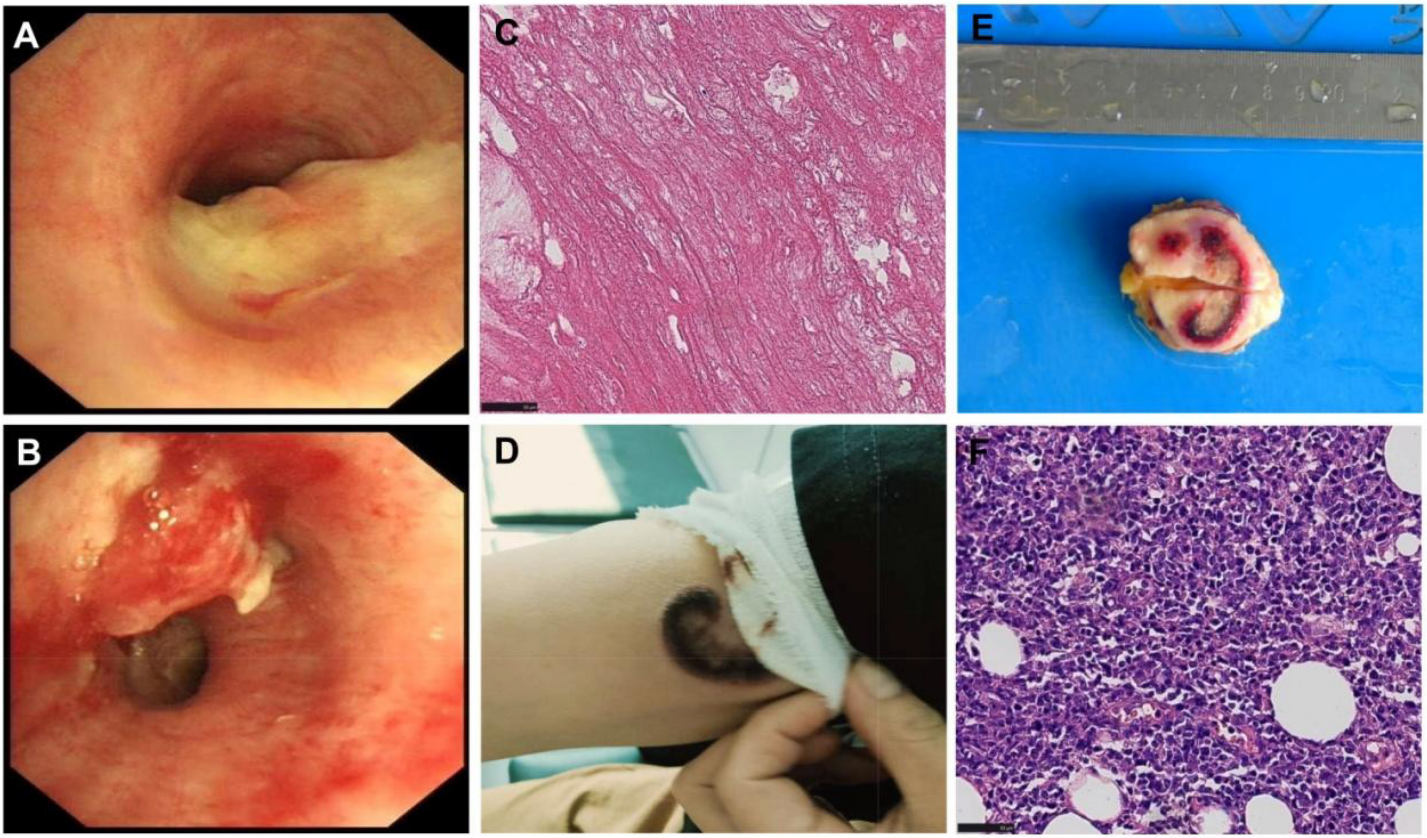
Bronchoscopy and tissue biopsies at admission. **(A,B)** Bronchoscope images. **(C)** Under the microscope, there are fibrinoid necrotic substances with scattered lymphocyte infiltration. 400× HE. **(D,E)** Photographs of a grayish-brown plaque on the right upper arm **(D)** before and **(E)** after excision. **(F)** Hyperplasia of dermis and subdermal fibrous tissue, proliferation of small blood vessels, numerous lymphoid-like cells distributed in clusters, accompanied by fat infiltration. The cells are of medium size, with an increased nuclear-cytoplasmic ratio. 400× HE.

Computed tomography of the chest showed left upper lobe consolidation with atelectasis; multiple, patchy, and bilateral opacities, nodules and masses; and a peripheral halo sign (Figure 1). Fibrobronchoscopy revealed a neoplasm obstructing the lumen of the left main bronchus (Figures 2A, B). BALF contained a normal level of galactomannan and did not contain malignant cells. Next-generation sequencing in BALF indicated the presence of *C. tropicalis* (sequence count: 3305). Histology of the left main bronchus neoplasm indicated necrotic fibrinous tissue containing isolated, atypically proliferating squamous cells (Figure 2C). The tissue was negative for *Mycobacterium tuberculosis* based on a PCR test.

We therefore diagnosed the patient with tropical candidal pneumonia and tracheobronchitis, with no evidence of lymphoma recurrence. The severity of her pulmonary infection led us to initiate antifungal treatment with intravenous caspofungin acetate (50 mg once daily). After 6 days of this treatment, bronchoscopy revealed a markedly smaller airway neoplasm.

During our efforts to understand what might have caused the immunosuppression that facilitated *C. tropicalis* infection, we discovered a palpable, well-defined, grayish-brown plaque measuring 3.2 cm × 0.8 cm on the medial aspect of the right upper arm (Figures 2D, E), which was not associated with substantial significant pain or itching. Histopathology confirmed that the plaque was malignant (Figure 2F).

**FIGURE 3:**
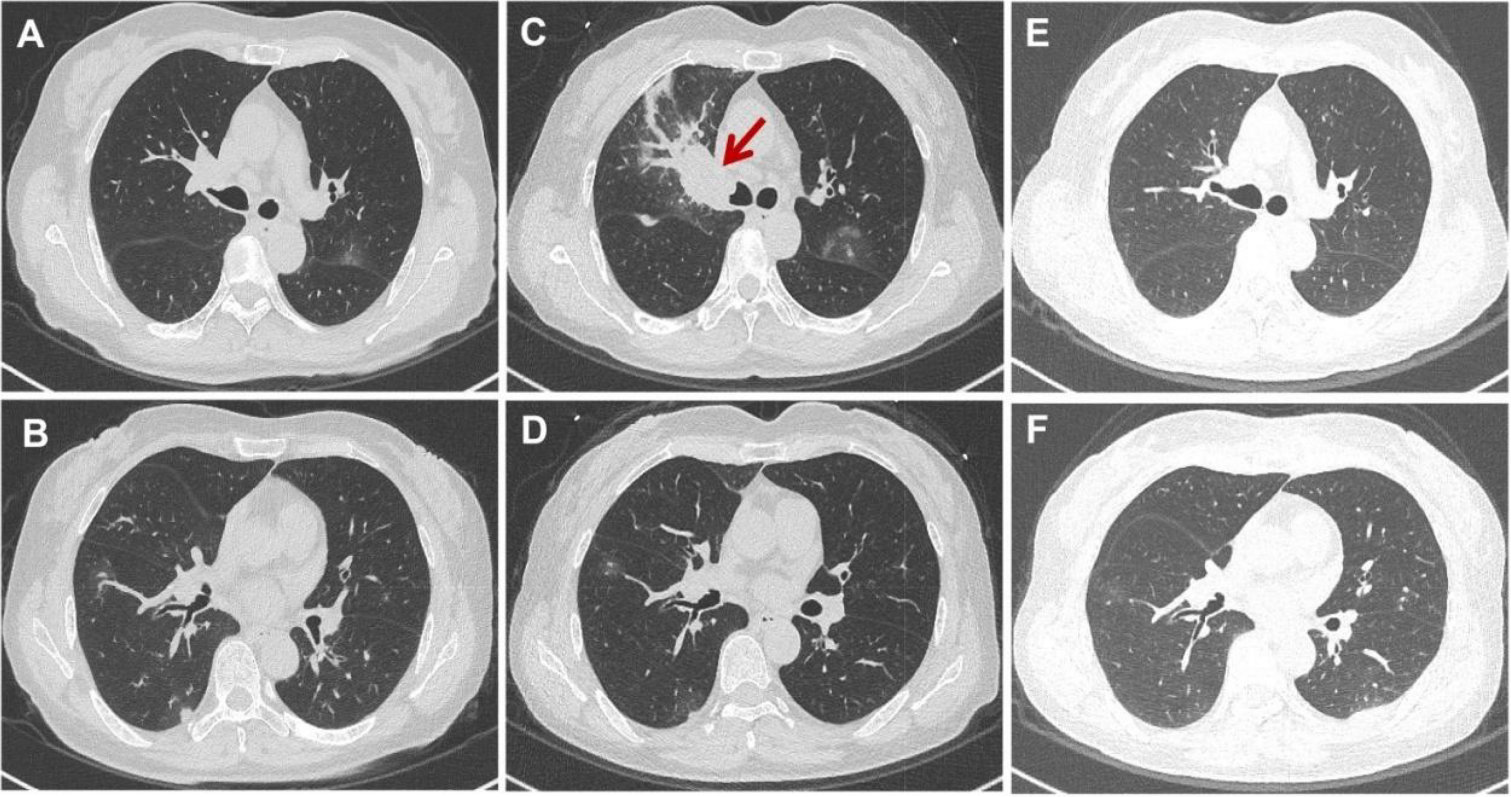
Computed tomography of the chest at different stages of treatment. **(A,B)** After 10 days of treatment with caspofungin acetate. The lung showed less consolidation and fewer nodules than at admission. **(C,D)** After 3 weeks on oral fluconazole. The lung showed new nodules and localized bronchial obstruction in the right upper lobe (red arrow) relative to admission. **(E,F)** After 4 weeks on caspofungin. The lung showed marked absorption and reduction of pulmonary lesions.

After 10 days on caspofungin acetate, computed tomography of the chest showed a recruitment maneuver on the left upper lobe and less nodularity in both lungs (Figures 3A, B). The patient was switched to oral fluconazole (400 mg once daily) and discharged. Three weeks after discharge, computed tomography of the chest revealed new pulmonary nodules and masses, including localized bronchial obstruction in the right upper lobe (Figures 3C, D).

We suspected fluconazole resistance, so the patient was switched to caspofungin (50 mg once daily). After 4 weeks on this regimen, computed tomography showed marked absorption and reduction of pulmonary lesions, and the original right hilar lesion was no longer visible (Figures 3E, F). The patient continues to be followed up. Written informed consent was obtained from the patient for publication of this case report and accompanying images.

## Discussion

The incidence of invasive fungal infections has been increasing among diverse patient populations for various reasons (5, 6), and those who are immunocompromised are at particularly high risk. In fact, the individual’s immune status remains a key determinant of how fungal infections of the lungs manifest and progress (7). Our patient had stopped immunosuppressive therapy to treat lymphoma for longer than 2 years before coming to our hospital, so we suspect that her cutaneous malignancy had compromised immune function and thereby facilitated her *C. tropicalis* infection.

We further speculate that *C. tropicalis* infection in our patient, in contrast to more frequent infection *via* the bloodstream (2), occurred primarily in the oropharynx and upper respiratory tract, from which it spread to the lower respiratory tract by virtue of the patient’s immunocompromised state. Nevertheless, it did not spread to other sites in the body, in contrast to frequently observed secondary infections affecting the eye, bones, joints, liver and spleen (8).

Although *C. tropicalis* is an opportunistic pathogen ubiquitous on the skin, mucous membranes and digestive tract, it rarely causes IPFD, which instead is due more often to *Aspergillus* species, *Cryptococcus neoformans*, *Pneumocystis jirovecii* and occasionally *C. albicans* (9, 10). This may help explain why the other hospital failed to diagnose the patient correctly.

The imaging findings of our patient were consistent with pneumonia associated with *C. tropicalis* or other *Candida* species, which is associated with nodules in 88%–95% of cases, consolidation in 50%–65%, and the halo and reversed halo sign in 33% (11), However, we also initially considered invasive pulmonary aspergillosis because this condition frequently presents as consolidation, nodules or masses on imaging (3), and it can present as airway stenosis and airway neoplasms (12). Our patient showed so-called “diffuse” bronchitis, in which pseudomembranous expansion extends beyond two tracheobronchial bifurcations through processes linked to immunosuppression (13). Such bronchitis contrasts with “localized” bronchitis, in which nodules, masses, pseudomembranes, or tracheobronchial necrosis do not extend beyond two tracheobronchial bifurcations. The characteristics of our patient suggest that diffuse bronchitis in patients with *C. tropicalis*-associated IPFD may be linked to antifungal resistance. Our patient did not present the ulcers, pseudomembranes, plaques or necrosis that affect a substantial proportion of individuals with candidal tracheobronchitis (14).

In addition to clinical and imaging findings, our diagnosis of the patient was based on the detection of *C. tropicalis* in sputum culture and next-generation sequencing of BALF. At the same time, histology of lung biopsies allowed us to exclude malignancy and *M. tuberculosis* infection as causes of the airway neoplasm. Our sequencing-supported diagnostic approach may avoid the disadvantages of more traditional approaches (4, 15, 16). *Candida* pneumonia can be diagnosed from biopsies based on the presence of abundant pseudohyphae, budding cells and/or spores as well as signs of inflammation (17), but such signs are present in only 30%–50% of people with invasive candidiasis (18). We failed to detect galactomannan in BALF, but this is not entirely surprising given the relatively low sensitivity of this assay (19, 20). Next-generation sequencing can be superior to PCR for molecular diagnosis because it can reveal infection even by novel pathogens that would not be detected using standard PCR primers (21).

The patient was presented at our hospital primarily with cough, expectoration and fever, even though fever is relatively uncommon among those with invasive fungal pneumonia who show normal levels of lymphocytes and monocytes (22). At the same time, her peripheral oxygenation, blood pressure, mental status and major organ function were normal. In principle, therefore, we considered the patient to have a mild, non-neutropenic fungal infection, for which first-line treatment would normally be azole-based medications (21). However, her lungs contained multi-lobar infiltrates with airway involvement. We therefore initiated antifungal therapy with intravenous caspofungin, typically given for severe fungal infections (23). This led to substantial clinical and radiological improvement, so the patient was switched to oral fluconazole therapy at discharge. This led her condition to worsen, leading us to suspect resistance. Therefore, we switched her to caspofungin, an echinocandin-based therapy typical against severe fungal infections (23).

The resistance in our patient is not surprising given that 7%–43% of *C. tropicalis* isolates from patients in several countries are resistant to fluconazole (24). Between December 2011 and December 2021 in China, the rate of *C. tropicalis* resistance to azoles rose from 5.7% to 31.8% in the case of fluconazole and from 5.7% to 29.1% in the case of voriconazole (1), and these rates are likely to increase given the high use of azole drugs in the country (25). Numerous contributors to such resistance have been identified, including mutations that weaken binding of azoles to the target enzyme ERG11 or mutations that upregulate ERG11 or the drug efflux pumps MDR1 and CDR1 (26–28). Another contributor to resistance is biofilm formation (29).

## Limitations

This case has the following limitations. Firstly, although the patient’s imaging condition deteriorated during the maintenance treatment with fluconazole, and improved significantly after switching to caspofungin, this suggests the possibility of *C. tropicalis* resistance to fluconazole. However, since no *in vitro* antifungal drug susceptibility testing (AST) was conducted on the isolated *C. tropicalis* strains, we cannot confirm the mechanism and spectrum of resistance at the microbiological level. Therefore, the conclusion of “azole resistance” in this article is more based on clinical efficacy inference rather than laboratory-confirmed “microbiological resistance.” Secondly, although we hypothesized that skin malignancy might be the potential cause of the patient’s impaired immune function, a systematic assessment of immune function (such as immune cells counts, *Candida tropicalis* specific IgG antibodies levels, CD4+ T-cell count, etc.) was not performed. Therefore, this assumption lacks direct immunological evidence support. Lastly, as a case report, the conclusion of this study has limited extrapolation. More cases accumulation and prospective studies are still needed to further clarify the clinical characteristics, resistance status, and host susceptibility factors of invasive pulmonary fungal disease caused by *C. tropicalis*.

## Conclusion

We report a rare case of IPFD that was caused by *C. tropicalis* and presented primarily as an airway neoplasm that led to initial misdiagnosis. The patient proved resistant to maintenance therapy with fluconazole but responded well to caspofungin. An undiagnosed cutaneous malignancy emerged as the likely cause of the immunosuppression that facilitated *C. tropicalis* infection. Our case underscores the diverse pulmonary presentations of *C. tropicalis*-associated IPFD, the need to exclude malignancies when the disease involves airway neoplasms, and the usefulness of exploring potential underlying causes of immunosuppression that may have facilitated *C. tropicalis* infection. Our case also highlights the need to monitor patients for azole resistance carefully, we call for that in the future, routine antifungal drug susceptibility testing (AST) should be conducted to obtain accurate data on drug resistance, and adjust treatment in a timely manner.

## Data Availability

The original contributions presented in the study are included in the article/supplementary material, further inquiries can be directed to the corresponding author.
